# Province-Level Prevalence of Psychiatric Disorders: Application of Small-Area Methodology to the Iranian Mental Health Survey (IranMHS)

**Published:** 2019-01

**Authors:** Farhad Moradpour, Ahmad Hajebi, Masoud Salehi, Masoud Solaymani-Dodaran, Afarin Rahimi-Movaghar, Vandad Sharifi, Masoumeh Amin-Esmaeili, Seyed Abbas Motevalian

**Affiliations:** 1 Department of Epidemiology, School of Public Health, Iran University of Medical Sciences, Tehran, Iran.; 2 Department of Psychiatry, Research Center for Addiction & Risky Behaviors, Iran University of Medical Sciences, Tehran, Iran.; 3 Department of Biostatistics, School of Public Health, Iran University of Medical Sciences, Tehran, Iran.; 4 Iranian National Center for Addiction Studies, Iranian Institute for Reduction of High-Risk Behaviors, Tehran University of Medical Sciences, Tehran, Iran.; 5 Department of Psychiatry, School of Medicine, Tehran University of Medical Sciences, Tehran, Iran.

**Keywords:** *Composite International Diagnostic Interview*, *Hierarchical Bayesian Model*, *Iran*, *Mental Disorders*, *Prevalence*, *Province-Level*, *Small Area Estimation*

## Abstract

**Objective:** National surveys revealed a high prevalence of psychiatric disorders in Iran. Province-level estimates are needed to manage the resources and focus on preventive efforts more efficiently. The objective of this study was to provide province-level estimates of psychiatric disorders.

**Method** **:** In this study, Iranian Mental Health Survey (IranMHS) data (n = 7886) was used to produce province-level prevalence estimates of any psychiatric disorders among 15-64 year old males and females. Psychiatric disorders were diagnosed based on structured diagnostic interview of the Persian version of Composite International Diagnostic Interview (CIDI, version, 2.1). The Hierarchical Bayesian (HB) random effect model was used to calculate the estimates. The mental health status of half of the participants was also measured using a 28-item general health questionnaire (GHQ).

**Results: **A wide variation in the prevalence of psychiatric disorders was found among 31 provinces of Iran. The direct estimates ranged from 3.6% to 62.6%, while the HB estimates ranged from 12.6% to 36.5%. The provincial prevalence among men ranged from 11.9% to 34.5%, while it ranged from 18.4% to 38.8% among women. The Pearson correlation coefficient between HB estimates and GHQ scores was 0.73.

**Conclusion: **The Bayesian small area estimation provides estimation with improved precision at local levels. Detecting high-priority communities with small-area approach could lead to a better distribution of limited facilities and more effective mental health interventions.

Psychiatric and substance use disorders are considered as one of the greatest public health concerns in both developed and developing countries. The global lifetime prevalence of common mental disorders (mood, anxiety, and substance use) in adults is 29.2% and the 12-month prevalence is estimated as 17.6% (1). Mental illnesses are also known as leading causes of the global burden of disease (GBD): mental disorders accounted for 6.8% of global disability-adjusted life years (DALYs) and 18.7% of global years lived with disability (YLDs) in GBD 2016(2, 3). 

In Iran, the burden of mental illnesses is ranked second after injuries (4). Substance use, depressive disorders, anxiety disorders, and bipolar disorder fall in 20 top causes of burden of disease in Iranian population (3). 

The prevalence of psychiatric disorders was reported from 17.1% to 23.6% in large national studies conducted in Iran (5-8).

Access to epidemiological information, such as prevalence, incidence, and risk factors are key components of decision-making and understanding the health status of communities (9).

Prevalence is one of the indicators for policy-making, budgeting, intervention, and modification of risk factors, especially for a subgroup of the population, such as social groups, or geographically defined areas, such as provinces or counties (10). 

Generally, national health surveys are designed to produce reliable direct estimates of the target population at national levels. Therefore, direct design-based surveys do not have sufficient power to provide reliable estimates at smaller subnational level, geographic domain, or groups formed by cross-classification of sociodemographic variables, which usually have a smaller sample size (11). 

While there are remarkable studies on estimation of the burden and prevalence of mental disorders at global, regional, and country levels (7, 12-13), few or even no reliable estimates are available for most countries at the subnational level. Local health care agencies and community health organizations do not have enough resources to collect data on their own and, thus, estimating such aspect of health seems more difficult and time-consuming, especially for non-fatal conditions such as mental disorders (14, 15).

To cope with the instability of the design-based direct estimation and provide estimates with improved precision, methods of Small-Area Estimation (SAE) that borrow strength from an auxiliary information, both at area or individual levels, have been increasingly considered (16). 

The general objective of this study was to estimate a 12-month prevalence of province-level psychiatric disorders using data of an Iranian mental health survey (IranMHS) (17).

## Materials and Methods

We used a small area method to provide the most reliable prevalence of mental disorders at province-level in the Iranian population. 

***Data Sources***

Two sources of data were used in this study: the individual-level data were provided by the Mental Health Office of Ministry of Health and the area-level data were obtained from the 2011 census of the Statistical Center of Iran.

***Individual Data***

The individual-level data consisted of the items collected as part of IranMHS, a national household cross-sectional study conducted in 2011 (17). IranMHS consisted of 7886 individuals aged 15-64 years who participated in a set of face to face interviews. The central measures of mental disorders used in this study were any persons who were diagnosed as mentally ill during the past 12-months, according to the criteria in Diagnostic and Statistical Manual of Mental Disorders, fourth edition (DSM-IV TR). Psychiatric disorders were diagnosed based on structured diagnostic interview of the Persian version of Composite International Diagnostic Interview (CIDI, version, 2.1): any anxiety disorder including panic disorder with/ without agoraphobia, agoraphobia without panic, social phobia, generalized anxiety disorder, obsessive-compulsive disorder, posttraumatic disorder; any mood disorder including major depressive disorder, dysthymia, bipolar 1 disorder; any substance use disorder including drug abuse/ dependence, alcohol abuse/ dependence; and any primary psychotic disorder. Finally, the dependent variable was coded as binary variable of the persons who had any of the aforementioned disorders.

The predictor variables were selected based on the experts’ opinions and previous literature (18) as well as availability of their parallel categorical form for extraction from 2011 census data. The variables included in the initial analytical dataset were gender, age, place of residence, occupation, education, income, and marital status. 

***Area-level Data***

Whenever necessary, the mentioned-classification was applied to the data obtained from 2011 census. This was done by adding up the numbers of individuals aged 15-64 years in each province based on different variables, and the total number was then expressed as a proportion of the whole population of the intended variable. 

***Estimation***

***Hierarchical Bayesian Model***

A multiple logistic regression with application of the complex sampling design was used to select the predictor to be included in the Bayesian model. Type 1 error; α = 0.1 was used as a significant level to select individual predictors (Table 2). 

To provide area-level prevalence of psychiatric disorders, a two-step small-area estimation was performed based on Malec et al. (19) with a Bayesian approach. Applying this mixed model provided a series of parameters with fixed effects that were common to all provinces, and a series of parameters with random effect which were specific for each area. First, a hierarchical Bayesian version of random-effect logit normal with both unit-level and area-level covariate was fitted to estimate the relationship between the predictor and the response variable “psychiatric disorder”. In the second step, an area-level logit model was performed based on previously measured relationship and the set of sociodemographic province-level covariate. 

The direct prevalence of psychiatric disorder was computed using weighted IranMHS 2011 data for all provinces. An estimation was also done by applying the Post-stratified Synthetic (PsSyn) and composite approaches (16). Reliability and precision of 3 small area methods, including post-stratified synthetic, composite, and hierarchical Bayesian model, were compared versus direct method.

***Evaluation of Province-level Estimates***

Due to the lack of a gold standard in Iran, a regional direct estimate was used as a reference to evaluate the validity of small-area province-level measurements (20). The province-level estimates, provided from the small-area analysis, were aggregated to 8 region-levels. The region-level prevalence estimates of mental disorders were directly obtained from IranMHS. The direct estimate at the regional level increased the sample size to the effective level and, therefore, provided stable and reliable measures of mental disorders. However, the region-level direct estimates were compared with the region-level aggregated small-area estimates. Then, discrepancy measures (15), such as mean square error (MSE), mean absolute difference (MAD), mean relative absolute difference (MRAD), rank statistics (RS), and correlation coefficient (CC), were calculated. MSE and confidence interval of prevalence for each small area were also estimated based on Rao 2003. 

In IranMHS, the mental health status of half of the participants was measured using the 28-item General Health Questionnaire (GHQ-28). The self-administrated GHQ-28 questionnaire was completed by the participants independently of the CIDI. The correlation between the estimated small-area prevalence and the mean score of GHQ for each province was estimated as another measure to verify the validity of the estimates. 

***Statistical Analysis***

R statistical software version 3.4 was used for programming direct estimation and design-based SAE. The hierarchical Bayesian analysis was performed by open BUGS, and 25 000 iterations with 5000 burn-in were monitored to produce all posterior distributions. The point prevalence map by province was produced using ArcGIS version 10.4, and Jenks natural break method was used to classify the data into different categories. 

## Results

In 2011, Iran Mental Health Survey (IranMHS) sampled 7886 individuals aged 15-64 years. It was previously shown that 23.6% of the general population was diagnosed with one type of mental disorder based on the DSM-IV criteria during the previous 12 months (7). 

Most provinces did not have adequate sample size to directly provide the prevalence of mental disorders. Nearly 84% (26 of 31) of the provinces had less than 450 observations. This is the minimum number of samples to provide stable estimates of mental disorders in the general population (1). Table 4, Appendix 1, provides detailed information on the number of provinces and their sample size of adults aged 15-64 years in IranMHS.

There was significant variation in the prevalence proportion of mental disorders among the Iranian population based on different demographic and socioeconomic characteristics. Females were more likely to be at risk than males, with a risk of 26.5% compared to 20.8%, respectively. The risk was dropping by about 4.8% at age 60-64 in comparison with younger ages. The detailed information about demographic and socioeconomic distribution of IranMHS and 2011 census and its corresponding prevalence has is presented in Table 1. 

The odds ratio of psychiatric disorders was found to be statistically significant for five variables out of seven. Sex, residence, marital status, occupation, and education were the predictors that had a p-value of less than 0.05 in the adjusted logistic regression model (Table 2). However, when the model was fitted separately for men and women, living in rural areas was significantly protective only for men (OR = 0.68). In contrast, higher education level was found to significantly affect mental disorders in women (OR = 0.73).

Finding of this study presented a large variation in the prevalence of mental disorders among 31 provinces of Iran. The prevalence of psychiatric disorders by direct estimates ranged from 3.6% to 62.6%, with a median of 23.3%. The 95% confidence interval (CI) width ranged from 6.2% to 34.3%, with a median of 14.8% (see table 5 appendix 2). The PsSyn had a narrow range from 23.3% to 24.3%, with a median of 23.7%; however, its 95% CIs width was very wide from 9.6% to 82.2%, with a median of 20.6% (Table 6, Appendix 2). The prevalence proportions estimated from composite (Table 7, Appendix 2) and hierarchical bayesian method (Table 8, Appendix 2) ranged from 17.0% to 36.4% and 12.6% to 36.5%, respectively, and their corresponding medians were 23.6% and 23%, respectively. The 95% CIs width for a composite estimate ranged from 6.2% to 100.0%, with a median of 13.7%, and it ranged from 5.6% to 22.0% for hierarchical Bayes estimates, with a median of 11.3%. The provincial prevalence among men ranged from 11.9% to 34.5%, with a median of 20.1% and confidence interval ranged from 7.3% to 27.2%. Among women, it ranged from 18.4% to 38.8%, with a median of 26.5% and confidence interval ranged from 8.1% to 25.9%. 

**Graph 1:**
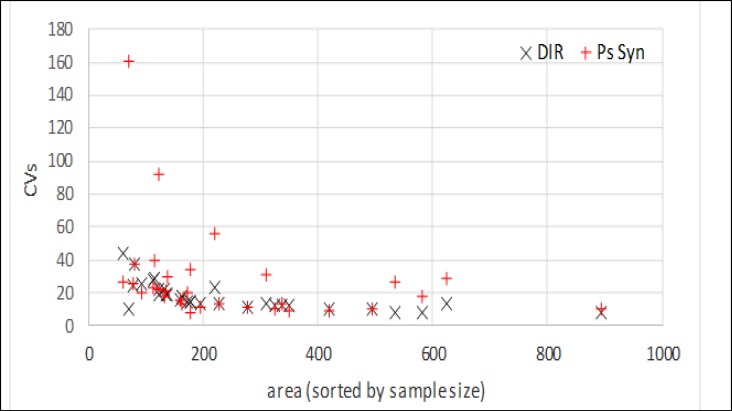
CVs of Poststratify Synthetic (PsSyn) and Direct (DIR) Estimator for each Area. Area Are Sorted by Increasing Sample Size

**Graph 2:**
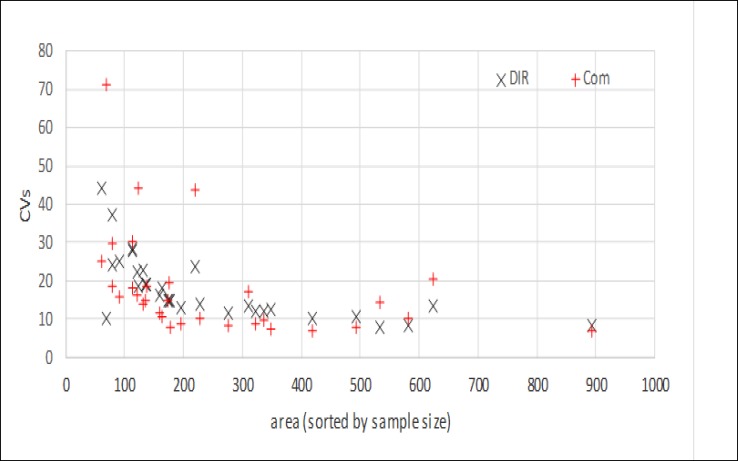
CVs of Composite (Com) and Direct (DIR) Estimator for each Area. Area Are Sorted by Increasing Sample Size

**Graph 3:**
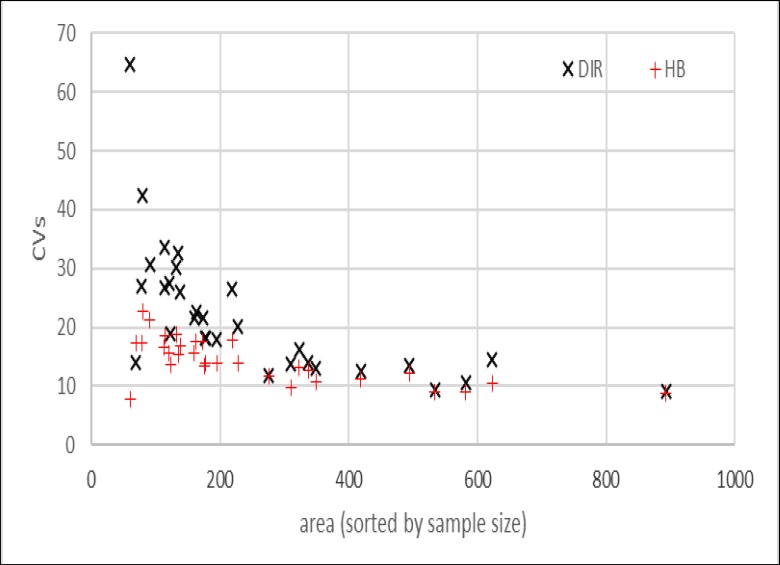
CVs of Hierarchical Bayes (HB) and Direct (DIR) Estimator for each Area. Area Are Sorted by Increasig Sample Siza

The area-level MSEs obtained from PsSyn did not show any improvement over the direct method. Moreover, the composite approach had no important improvement compared to the direct approach (Table 5-7, Appendix 2). However, values estimated by HB improved considerably compared to the direct method for all provinces (Table 8, Appendix 2). Graphs 1-3, Appendix 3 present the scatter plot of coefficient of variation (CV) versus increasing sample size for each method versus direct estimates. The HB also shows a smaller CVs, especially for smaller sample sizes on the left of the graph. 

The 8 region aggregated prevalence of psychiatric disorders was compared to the corresponding direct region-level reference estimates. The PsSyn approach produced the largest discrepancy statistics and did not show any improvement over the direct estimates (Table 3). HB was the best small-area technique for estimating the prevalence of mental disorders per province. The CC was close to 1, and all other 4 discrepancy statistics were closest to 0 (Table 3).

After removing the outliers, the correlation coefficients between the mean score of GHQ-28 and parallel prevalence estimates of psychiatric disorders were 0.57 (Direct), 0.03 (PsSyn), 0.58 (Composite), and 0.73 (HB), respectively.

**Figure 1:**
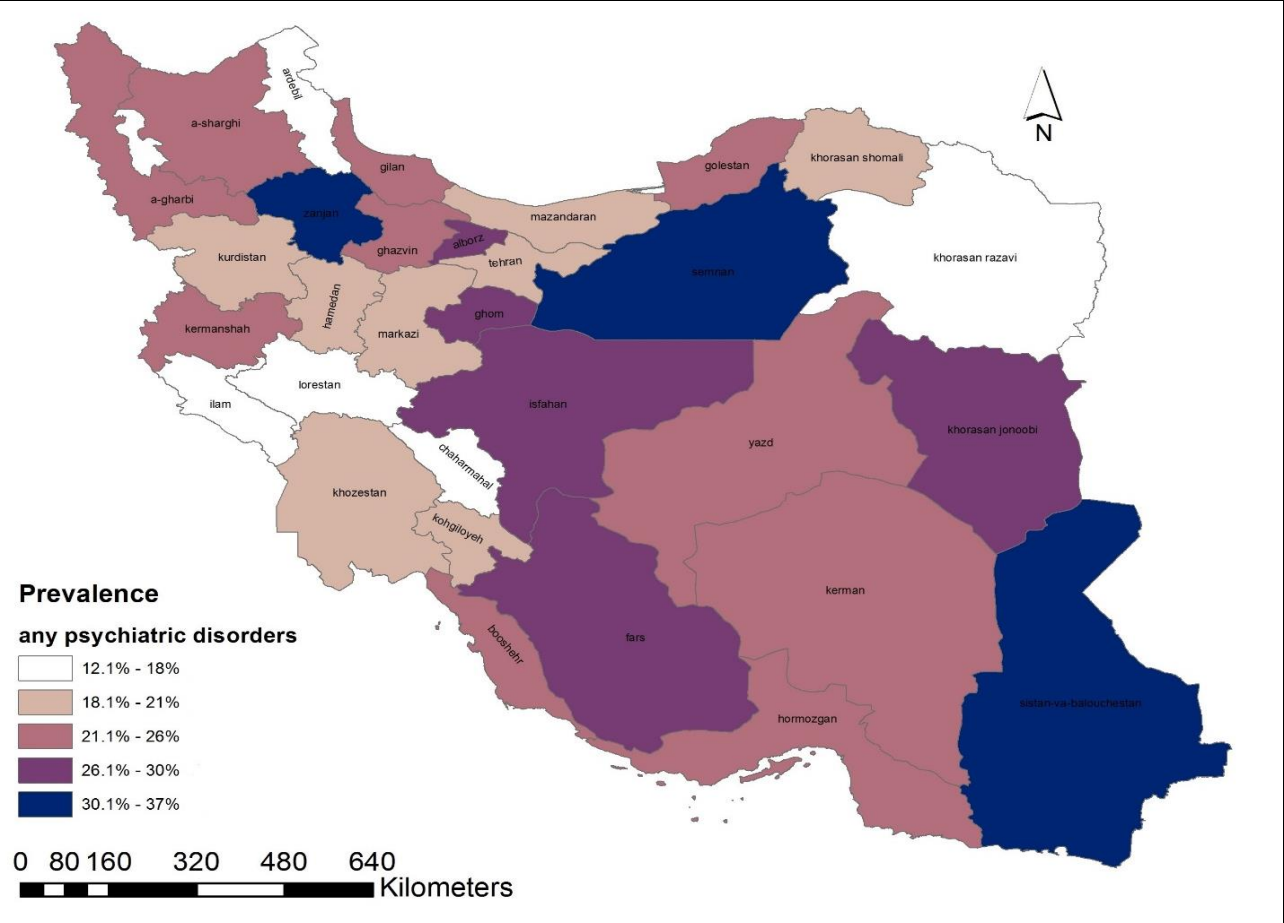
Province-Level Prevalence of any Psychiatric Disorder Aged 15-64 Years, Obtained from Hierarchical Bayesian Random Effect Model

**Figure 2:**
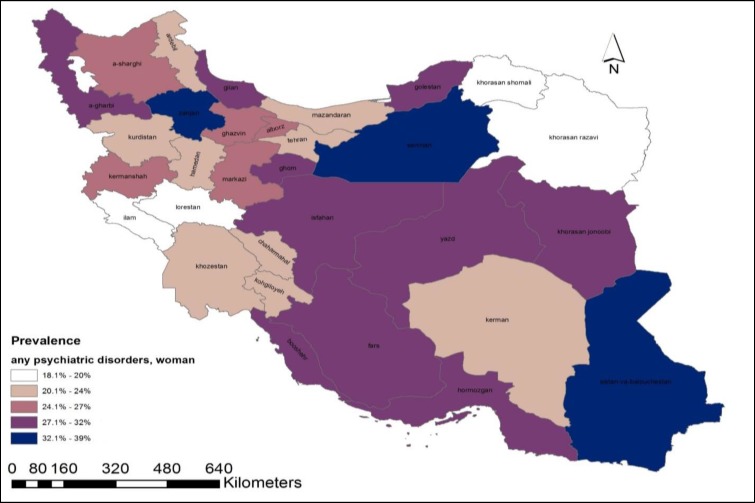
Province-Level Prevalence of any Psychiatric Disorder in Women Aged 15-64 Years, Obtained from Hierarchical Bayesian Random Effect Model

**Figure 3:**
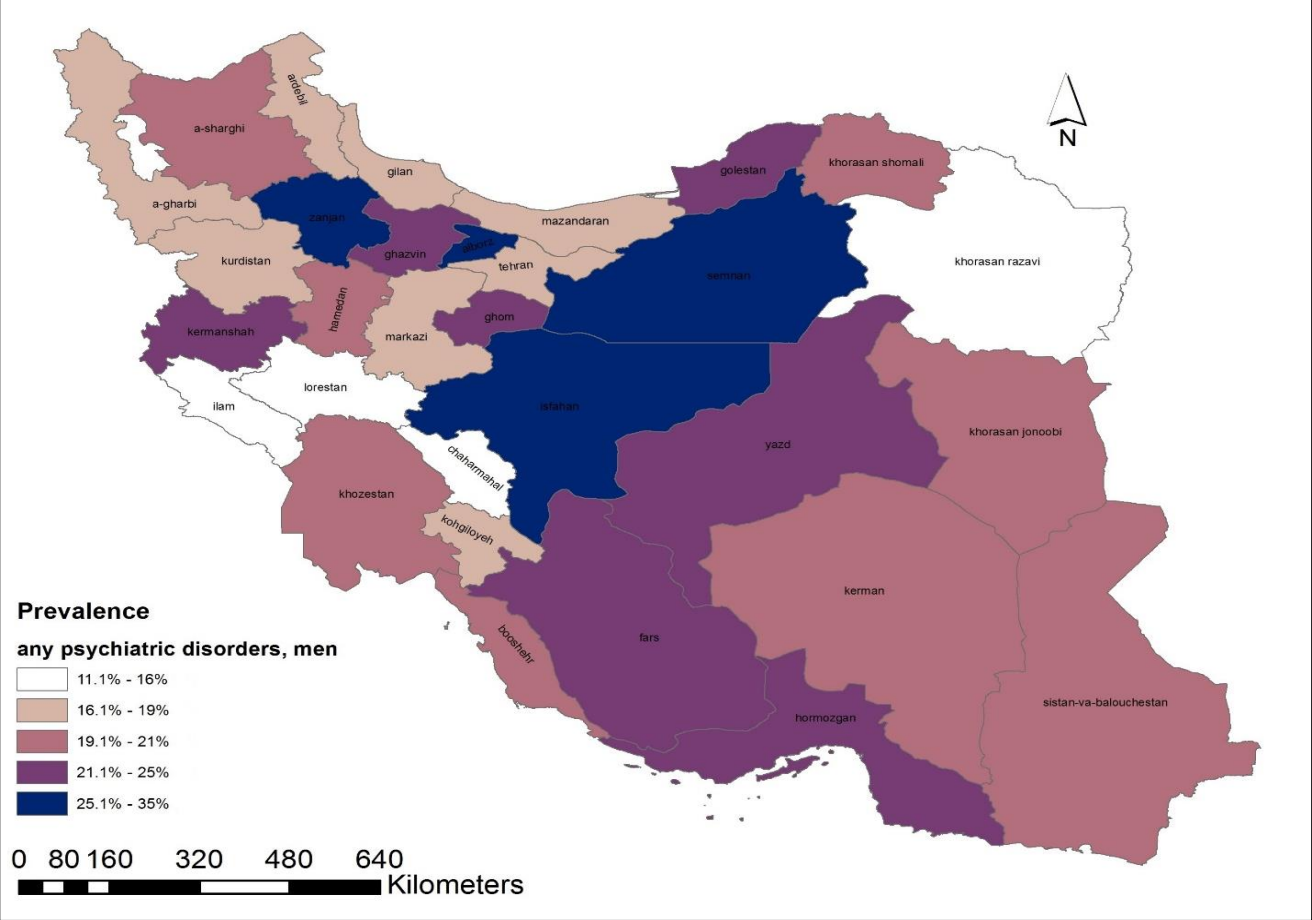
Province-Level Prevalence of any Psychiatric Disorder in Men Aged 15-64 Years, Obtained from Hierarchical Bayesian Random Effect Model

The HB estimates of mental disorders were highly varied by geographic location and sex of the Iranian population. This diversity was presented as a geographical map of point prevalence of mental disorders (Figure 1-3). According to the maps, provinces can be classified as 3 prevalence regions: low prevalence (white and gray), moderate prevalence (pink), and high prevalence (purple and dark navy) of mental disorders. The provinces with the lowest proportion were found in the west, southwest, north, and northeast regions. Low-prevalence provinces were like a belt beginning from the west and southwest of Iran and going across a line to the Northern provinces and the margin of the Caspian Sea. The provinces with higher prevalence were located in the center, southeast, and some southern regions of Alborz mountains. 

**Table 1:** Descriptive Statistics on Demographic and Socioeconomic Predictors of IranMHS and 2011 Census (Study Sample Size = 7886)

**Predictor**	**n**	**Unweighted ** **Proportion (%)**	**Weighted ** **Proportion (%)**	**Proportion in the ** **census 2011 (%)**	**Mental disorder ** **proportion (%)**
Gender					
Female	4,499	57.1	49.5	26470236 (49.7)	26.5
Male	3,387	42.9	50.5	26737555 (50.3)	20.8
Residence					
Urban	4,380	55.5	70.9	38761947 ( 72.9)	24.2
Rural	3,506	44.5	29.1	14445844 (27.1)	22.1
Age					
15-19	998	12.7	18.1	6607043 (13.9)	21.4
20-29	2549	32.3	33.8	17087151 (33)	24.6
30-39	2200	27.9	21.8	12542942 (22)	23.9
40-49	1188	15.0	15.3	8937230 (16)	24.5
50-59	704	9.0	8.7	6207527 (11.6)	23.4
60-64	247	3.1	2.5	1861503 (3.5)	18.9
Marital					
Never married	2,025	25.7	32.9	15940696 (30)	22.8
Married	5,527	70.1	63.5	35427355 (66.6)	23.3
Previously-married	332	4.2	3.6	1839738 (3.4)	36.1
Education					
Illiterate	646	8.2	5.7	3301177 (6.2)	26.4
Primary	1917	24.4	19.6	10223001(19.2)	25.9
Secondary	1280	16.3	15.5	7773740(14.6)	25.1
High school	2823	35.8	40.7	21830368(40.1)	23.3
University	1,208	15.3	18.5	10063267 (18.9)	20.1
Income (Rials)					
<=5000000	6,218	79.5	74.3	*18491 (48.3)	24.6
>5000000	1,599	20.5	25.6	*19794 (51.7)	20.4
Unemployment					
Yes	737	9.3	9.5	3532070 (6.6)	33.0
No	7,149	90.6	90.5	49712730 (93.4)	22.6

IranMHS: Iranian Mental Health Survey

* Estimated From Iranian Household Expenditure and Income 2011

**Table 2:** Multivariable Logistic Model of Mental Illness Regressed on Demographic and Socioeconomic Predictors (Sample Size = 7886)

	**Male**		**Female**		**Total**	
**Predictor**	**OR (95% CI)**	**P-value**	**OR (95% CI)**	**P-value**	**OR (95% CI)**	**P-value**
Gender						
Male	(*)	(*)	(*)	(*)	1	
Female	(*)	(*)	(*)	(*)	1.39 (1.22-1.59)	< 0.001
Residence						
Rural	1	(*)	1	(*)	1	(*)
Urban	1.47 (1.18-1.82)	0.001	1.10 (0.92-1.31)	0.309	1.28 (1.1-1.48)	0.001
Marital status						
Other	1	(*)	1	(*)	1	(*)
Married	1.006 (0.81-1.25)	0.952	1.06 (0.87-1.3)	0.536	1.04 (0.89-1.21)	0.587
Previously- married	3.4 (1.7-6.7)	< 0.001	1.48 (1.03-2.1)	0.032	1.75 (1.27-2.4)	0.001
Unemployment						
No	1	(*)	1	(*)	1	(*)
Yes	1.93 (1.48-2.52)	< 0.001	1.6 (1.16-2.28)	0.005	1.78 (1.45-2.2)	< 0.001
Education						
Illiterate	1	(*)	1	(*)	1	(*)
Primary	0.90 (0.51- 1.60)	0.730	1.02 (0.75- 1.38)	0.890	0.97 (0.74- 1.26)	0.813
Secondary	0.79 (0.43- 1.43)	0.437	1.15 (0.82- 1.62)	0.418	0.95 (0.71- 1.28)	0.760
High school	0.74 (0.42- 1.31)	0.297	0.94 (0.69- 1.29)	0.720	0.84 (0.64- 1.11)	0.220
University	0.64 (0.34-1.20)	0.163	0.73 (0.50 -0.99)	0.049	0.66 (0.48 - 0.91)	0.011
Income (Rials)						
>5000000	1	(*)	1	(*)	1	(*)
<=5000000	1.25 (0.96-1.64)	0.106	1.14 (0.92-1.43)	0.254	1.19 (0.99-1.4)	0.055
Age						
15-19	1	(*)	1	(*)	1	(*)
20-29	1.36 (0.95- 1.95)	0.093	1.11 (0.84- 1.48)	0.458	1.21 (0.96- 1.53)	0.094
30-39	1.31 (0.84- 2.03)	0.231	1.07 (0.78- 1.46)	0.672	1.15 (0.89- 1.45)	0.245
40-49	1.34 (0.83- 2.15)	0.230	1.02 (0.73- 1.44)	0.885	1.13 (0.85- 1.49)	0.406
50-59	0.95 (0.56- 1.66)	0.860	1.09 (0.72- 1.65)	0.670	0.99 (0.70- 1.38)	0.940
60-64	0.82 (0.34- 1.98)	0.666	0.71 (0.39- 1.28)	0.250	0.72 (0.43- 1.22)	0.227

**Table 3:** Discrepancy Measures Comparing Aggregated Small-Area Approaches and Direct Region-Level Estimates from IranMHS 2011

	**Total**				**Male**				**Female**			
**Correlation Coefficient**	**PsSyn**	**Composite**	**HB**	**Dir**	**Syn**	**Composite**	**HB**	**Dir**	**Syn**	**Composite**	**HB**	**Dir**
MSE*	-0.219	0.992	0.971	0.538	-0.003	0.961	0.931	0.483	-0.644	0.997	0.939	0.592
MAD*	0.023	0.020	0.017	0.017	0.018	0.016	0.015	0.015	0.028	0.024	0.020	0.022
MRAD*	0.031	0.018	0.013	0.043	0.024	0.014	0.011	0.041	0.038	0.023	0.018	0.045
RS*	0.143	0.087	0.052	0.179	0.126	0.075	0.054	0.194	0.160	0.097	0.069	0.168

* Values close to 0 indicate a better estimate

## Discussion

***Main Findings***

In this study, a small-area values of psychiatric disorders in the general population aged 15-64 years were estimated at the province-level. As depicted in the maps, the estimated prevalence of psychiatric disorders had a considerable diversity across provinces and genders. The variation might be due to the sociodemographic composition of communities, and not following a specific pattern in the provinces. It was also found that regionally accumulated small-area values were highly comparable with their corresponding direct estimates, which were set as reference values. 

Direct estimation had a great discrepancy statistics, which may be due to the fact that IranMHS was designed at the national scale; and when the sample was broken down to the subnational level, there was no longer enough power to provide stable statistics. The synthetic approach is widely used in the field of public health, perhaps because of its simplicity (15, 21-23). However, if the characteristics of a large area which covers local ones are not constant and similar to each other, this method is discouraged and biased (24). 

The HB method clearly produced lower values of MSEs at the province-level than the direct method. In the composite method, although the MSEs at the province-level presented lower values than the direct method, there were some fluctuations in their ventricles. Scattering of the CVs versus sample size by comparing each method with the direct approach also confirmed the accuracy of the HB method. Increased accuracy provided by HB was more impressive in small domains with sample sizes lower than 420 individuals. However, regarding the correlation with reference values and other discrepancy measures, HB was found to be preferred compared to the synthetic and composite methods. 

The HB analysis used in this study had one area-level random effect. According to the test of the variance, the equality of variance component (null hypothesis) for province-level random effect was not retained. Therefore, the estimates of random components were all far from 0 and had a significant effect on the prevalence estimates at the province-level. 

In this analysis, the spatial correlation of psychiatric disorders was estimated by means of calculating Moran index and its corresponding p-values, which were 0.15 and 0.29, respectively. Thus, the spatial random effect term was not included in the model because of very little impact on the corresponding prevalence estimation. 

***Previous Direct Studies***

No comprehensive subnational studies considered the prevalence of mental disorders in all provinces, and studies have been limited to specific populations. Based on the time scale, these studies can be divided into 2 pre-revolutionary periods, from 1962 to 1971, with a prevalence of 11.9% to 18.6%, and after the revolution, from 1992 to 1999, with a prevalence of 12.5% to 30.2% (25). According to Sharifi et al., among the studies conducted to estimate psychiatric illnesses during 1992 to 2005, the participants were randomly selected only in 43 studies. Furthermore, the characteristics of the samples under study were representatives of the target populations only in 40% of the randomly selected studies.

Three national surveys conducted in 1999(5), 2001(6), and 2011(7) reported psychiatric disorder prevalence of 21.0%, 17.1%, and 23.6%, respectively. Other studies have also examined mental health status in Iran (26). Their application was not intended for small areas or for use in other target populations, and other aspects, such as estimating the prevalence for care in the same population, were considered. 

A systematic review conducted in 2007 reported a wide range proportion of 1.9% to 58.8% for mental disorders (26). The median prevalence in this study was 18.6% and the mean prevalence 21.9%. The study of health and disease was conducted in 2008 on Iranian population aged 18 and over (27). The results of that study showed that Yazd, Mazandaran, and Ilam had the lowest prevalence of mental illnesses, respectively, and Chaharmahal-va-Bakhtiari, Golestan and Hamedan had the highest prevalence, respectively. According to the present study, Semnan, Zanjan, and Sistan-Va-Baluchestan remained at the top ranks in terms of mental illness prevalence. A recent nationwide study conducted by Norballa et al. (2015) used GHQ-28 as a screening tool and found the prevalence of 23.4% in the sample population of 15 years and older (8). They also reported a direct province-level prevalence of mental disorders with range of 12.8% in Golestan and 36.3% in Lorestan (28).

A national survey used different instruments for diagnostic and screening psychiatric disorders in Iran. Mohammadi et al. (2001) used schizophrenia and Affective Disorders Scale (SADS). The study conducted by Norballa et al. (1999, 2015) used the GHQ-28, and sharifi, et al. (2011) used CIDI diagnostic tool.

Studies conducted on mental disorders used different approaches, assessment tools, or even different study powers. Therefore, it is not reasonable to compare the results of these studies due to their difference in the use of tools and scoring method.

## Limitation

The methods used in this study were developed mostly outside the scope of health (29-30) and, recently, they have been used in the field of health as well as mental health (31-32). However, there are limitations to our study that should be considered when interpreting and generalizing the results. Although HB improved the estimate of mental disorder prevalence, its precision was still low due to relatively wide CIs. A solution to this problem could be the accumulation of more years of data collected through the same method. Another problem was the unavailability of area-level demographic data with the same precision and categories as the individual data. Since GHQ-28 is a screening tool, its province-level findings (33) could not be compared with CIDI-based diagnoses in this study. So, there was no available, reliable direct estimate as a gold standard for the evaluation of current SAE province-level results.

## Conclusion

The findings of this study on the indirect estimate of epidemiologic measures at the local level, suggested that in the absence of reliable direct estimates, specifically for small areas with lower sample sizes, hierarchical Bayesian SAE could add valuable information to the nationally-designed surveys. The results of this study might be useful for policy-making and local health network funding. Given the large variation between provincial estimates (about 2 times between the lowest and the highest), this finding might be considered a trusted source for distribution of mental health facilities. Communities with various prevalence of mental disorders have different risk factors. Such differences should be considered when designing studies. Thus, for conducting a study on mental disorders, the minimum sample size of small communities, such as counties or provinces, should not be less than 420. However, as a new application of sampling statistics, the SAE methodology needs to be more developed and its application should be further assessed in Iran.

**Appendix 1**

**Table 4:** Descriptive Statistics of the 2011 IranMHS Sample by Provinces

**Provinces**	**Male**		**Female**		**Total**	
	**n**	**Proportion (%)**	**n**	**Proportion (%)**	**n**	**Proportion (%)**
A-gharbi	170	5.02	179	3.98	349	4.43
A-sharghi	212	6.26	207	4.60	419	5.31
Alborz	72	2.13	123	2.73	195	2.470
Ardebil	61	1.80	71	1.58	132	1.670
Booshehr	49	1.45	64	1.42	113	1.430
Chaharmahal-va-bakhtiari	45	1.33	69	1.53	114	1.450
Fars	239	7.06	295	6.56	534	6.770
Ghazvin	73	2.16	65	1.44	138	1.750
Ghom	63	1.86	72	1.60	135	1.710
Gilan	109	3.22	167	3.71	276	3.500
Golestan	69	2.04	109	2.42	178	2.260
Hamedan	67	1.98	96	2.13	163	2.070
Hormozgan	76	2.24	100	2.22	176	2.230
Ilam	25	0.74	35	0.78	60	0.760
Isfahan	263	7.76	319	7.09	582	7.380
Kerman	124	3.66	199	4.42	323	4.100
Kermanshah	101	2.98	127	2.82	228	2.890
Khorasan jonoobi	35	1.03	43	0.96	78	0.990
Khorasan razavi	236	6.97	387	8.60	623	7.900
Khorasan shomali	38	1.12	53	1.18	91	1.150
Khozestan	236	6.97	258	5.73	494	6.260
Kohgiloyeh-va-boyer-ahmad	38	1.12	42	0.93	80	1.010
Kurdistan	95	2.80	78	1.73	173	2.190
Lorestan	102	3.01	117	2.60	219	2.780
Markazi	64	1.89	96	2.13	160	2.03
Mazandaran	111	3.28	226	5.02	337	4.27
Semnan	33	0.97	36	0.80	69	0.87
Sistan-va-balouchestan	132	3.90	178	3.96	310	3.93
Tehran	352	10.39	541	12.02	893	11.32
Yazd	47	1.39	74	1.64	121	1.53
Zanjan	50	1.48	73	1.62	123	1.56

**Appendix 2**

**Table 5:** Psychiatric Disorder Prevalence by Province, Direct Method Estimated from IranMHS 2011

**Province**	**Total**		**Male**		**Female**	
	**Proportion (95%CI)**	**MSE**	**Proportion (95% CI)**	**MSE**	**Proportion (95%CI)**	**MSE**
A-gharbi	0.245 (0.2-0.29)	0.00052	0.184 (0.135-0.182)	0.00071	0.314 (0.249-0.383)	0.00116
A-sharghi	0.224 (0.183-0.265)	0.00043	0.201 (0.154-0.2)	0.00065	0.255 (0.201-0.314)	0.00083
Alborz	0.277 (0.233-0.321)	0.00052	0.287 (0.204-0.285)	0.00213	0.269 (0.2-0.347)	0.00140
Ardebil	0.178 (0.122-0.234)	0.00081	0.177 (0.112-0.175)	0.00132	0.215 (0.141-0.3)	0.00163
Booshehr	0.234 (0.164-0.304)	0.00128	0.203 (0.129-0.2)	0.00168	0.273 (0.19-0.369)	0.00207
Chaharmahal- va-bakhtiari	0.166 (0.105-0.227)	0.00096	0.158 (0.09-0.157)	0.00143	0.215 (0.142-0.298)	0.00160
Fars	0.281 (0.243-0.319)	0.00038	0.25 (0.2-0.249)	0.00070	0.318 (0.263-0.377)	0.00084
Ghazvin	0.252 (0.185-0.319)	0.00118	0.233 (0.162-0.23)	0.00156	0.266 (0.184-0.359)	0.00198
Ghom	0.262 (0.192-0.332)	0.00127	0.227 (0.152-0.224)	0.00174	0.293 (0.211-0.386)	0.00202
Gilan	0.23 (0.182-0.278)	0.00060	0.175 (0.12-0.174)	0.00091	0.294 (0.229-0.363)	0.00117
Golestan	0.253 (0.194-0.312)	0.00092	0.237 (0.164-0.235)	0.00169	0.273 (0.203-0.351)	0.00142
Hamedan	0.201 (0.146-0.256)	0.00079	0.201 (0.134-0.199)	0.00138	0.202 (0.138-0.276)	0.00125
Hormozgan	0.258 (0.197-0.319)	0.00097	0.213 (0.146-0.211)	0.00139	0.299 (0.224-0.382)	0.00162
Ilam	0.175 (0.153-0.197)	0.00013	0.152 (0.076-0.15)	0.00179	0.187 (0.092-0.27)	0.00021
Isfahan	0.284 (0.246-0.322)	0.00037	0.27 (0.22-0.269)	0.00072	0.303 (0.252-0.358)	0.00074
Kerman	0.211 (0.173-0.249)	0.00037	0.21 (0.152-0.209)	0.00098	0.212 (0.16-0.27)	0.00079
Kermanshah	0.252 (0.205-0.299)	0.00058	0.246 (0.179-0.244)	0.00135	0.268 (0.199-0.344)	0.00137
Khorasan jonoobi	0.26 (0.176-0.344)	0.00182	0.203 (0.124-0.199)	0.00201	0.304 (0.209-0.415)	0.00278
Khorasan razavi	0.159 (0.13-0.188)	0.00022	0.139 (0.101-0.138)	0.00044	0.196 (0.157-0.237)	0.00042
Khorasan shomali	0.205 (0.138-0.272)	0.00117	0.206 (0.128-0.203)	0.00199	0.192 (0.117-0.278)	0.00168
Khozestan	0.195 (0.165-0.225)	0.00023	0.191 (0.146-0.19)	0.00058	0.218 (0.169-0.272)	0.00070
Kohgiloyeh- va-boyer- ahmad	0.184 (0.116-0.252)	0.00120	0.161 (0.092-0.159)	0.00149	0.202 (0.119-0.298)	0.00208
Kurdistan	0.188 (0.136-0.24)	0.00071	0.179 (0.119-0.178)	0.00104	0.228 (0.154-0.311)	0.00160
Lorestan	0.126 (0.084-0.168)	0.00047	0.119 (0.068-0.117)	0.00080	0.185 (0.124-0.252)	0.00108
Markazi	0.205 (0.152-0.258)	0.00072	0.188 (0.121-0.186)	0.00141	0.242 (0.172-0.318)	0.00142
Mazandaran	0.201 (0.162-0.24)	0.00039	0.183 (0.127-0.181)	0.00093	0.205 (0.157-0.259)	0.00069
Semnan	0.365 (0.254-0.476)	0.00321	0.345 (0.223-0.34)	0.00499	0.384 (0.265-0.524)	0.00443
Sistan-va balouchestan	0.3 (0.249-0.351)	0.00068	0.197 (0.142-0.196)	0.00088	0.378 (0.308-0.451)	0.00134
Tehran	0.194 (0.168-0.22)	0.00018	0.165 (0.131-0.165)	0.00034	0.238 (0.198-0.28)	0.00044
Yazd	0.248 (0.176-0.32)	0.00135	0.211 (0.133-0.208)	0.00196	0.289 (0.207-0.383)	0.00202
Zanjan	0.361 (0.28-0.442)	0.00170	0.274 (0.187-0.271)	0.00252	0.388 (0.29-0.495)	0.00276

**Table 6:** Psychiatric Disorder Prevalence by Province, Small Area Post-Stratified Synthetic Method

**Province**	**Total**		**Male**		**Female**	
	**Proportion (95% CI)**	**MSE**	**Proportion (95%CI)**	**MSE**	**Proportion (95% CI)**	**MSE**
A-gharbi	0.235 (0.194-0.275)	0.00043	0.205 (0.198-0.212)	0.00001	0.265 (0.076-0.454)	0.00930
A-sharghi	0.237 (0.195-0.278)	0.00044	0.208 (0.127-0.29)	0.00174	0.267 (0.184-0.349)	0.00177
Alborz	0.242 (0.186-0.298)	0.00081	0.217 (-0.032-0.467)	0.01624	0.267 (0.206-0.329)	0.00098
Ardebil	0.236 (0.155-0.317)	0.00171	0.205 (0.183-0.227)	0.00013	0.266 (0.149-0.383)	0.00357
Booshehr	0.236 (0.13-0.342)	0.00294	0.21 (0.112-0.309)	0.00253	0.265 (0.148-0.383)	0.00357
Chaharmahal- va-bakhtiari	0.234 (0.054-0.414)	0.00841	0.204 (0.048-0.36)	0.00634	0.265 (0.112-0.417)	0.00608
Fars	0.238 (0.117-0.36)	0.00385	0.211 (0.05-0.372)	0.00671	0.266 (0.061-0.47)	0.01089
Ghazvin	0.237 (0.097-0.378)	0.00514	0.209 (-0.012-0.429)	0.01268	0.266 (0.006-0.527)	0.01768
Ghom	0.241 (0.143-0.339)	0.00249	0.216 (0.099-0.332)	0.00355	0.267 (0.239-0.295)	0.00021
Gilan	0.237 (0.185-0.289)	0.00071	0.207 (0.184-0.23)	0.00014	0.266 (0.098-0.434)	0.00735
Golestan	0.234 (0.199-0.269)	0.00032	0.202 (0.029-0.375)	0.00780	0.265 (0.217-0.313)	0.00059
Hamedan	0.236 (0.174-0.298)	0.00099	0.206 (0.135-0.276)	0.00130	0.266 (0.196-0.337)	0.00130
Hormozgan	0.233 (0.079-0.387)	0.00616	0.202 (0.004-0.401)	0.01029	0.264 (0.035-0.494)	0.01371
Ilam	0.238 (-0.158-0.634)	0.04082	0.21 (-0.082-0.502)	0.02221	0.267 (-0.132-0.665)	0.04133
Isfahan	0.24 (0.158-0.322)	0.00177	0.214 (0.059-0.37)	0.00631	0.266 (0.117-0.415)	0.00577
Kerman	0.236 (0.188-0.283)	0.00059	0.206 (0.189-0.223)	0.00008	0.266 (0.192-0.339)	0.00139
Kermanshah	0.239 (0.175-0.304)	0.00109	0.212 (0.11-0.313)	0.00270	0.267 (0.221-0.313)	0.00055
Khorasan jonoobi	0.233 (0.118-0.348)	0.00345	0.201 (0.19-0.212)	0.00003	0.265 (0.156-0.374)	0.00309
Khorasan razavi	0.238 (0.102-0.373)	0.00481	0.208 (0.119-0.298)	0.00209	0.266 (0.156-0.377)	0.00320
Khorasan shomali	0.233 (0.142-0.323)	0.00213	0.199 (0.068-0.33)	0.00447	0.265 (0.109-0.422)	0.00636
Khozestan	0.237 (0.19-0.284)	0.00058	0.21 (0.197-0.223)	0.00005	0.265 (0.24-0.289)	0.00016
Kohgiloyeh- va-boyer- ahmad	0.236 (0.065-0.407)	0.00761	0.205 (0.119-0.291)	0.00192	0.265 (0.04-0.491)	0.01324
Kurdistan	0.236 (0.142-0.33)	0.00231	0.207 (0.149-0.264)	0.00086	0.266 (0.195-0.337)	0.00132
Lorestan	0.237 (-0.023-0.497)	0.01762	0.208 (-0.038-0.454)	0.01579	0.266 (0.076-0.455)	0.00936
Markazi	0.238 (0.165-0.311)	0.00139	0.21 (0.161-0.259)	0.00062	0.267 (0.23-0.304)	0.00035
Mazandaran	0.235 (0.173-0.296)	0.00099	0.204 (0.191-0.216)	0.00004	0.266 (0.225-0.307)	0.00044
Semnan	0.238 (-0.51-0.987)	0.14583	0.21 (-0.455-0.876)	0.11537	0.267 (-0.701-1.234)	0.24370
Sistan-va- balouchestan	0.233 (0.094-0.373)	0.00506	0.201 (0.181-0.221)	0.00010	0.265 (-0.08-0.609)	0.03092
Tehran	0.243 (0.197-0.289)	0.00055	0.22 (0.194-0.247)	0.00018	0.266 (0.221-0.312)	0.00055
Yazd	0.237 (0.135-0.34)	0.00274	0.21 (0.127-0.293)	0.00180	0.266 (0.105-0.428)	0.00679
Zanjan	0.235 (-0.188-0.658)	0.04659	0.204 (-0.22-0.628)	0.04679	0.265 (-0.32-0.851)	0.08921

**Table 7:** Psychiatric Disorder Prevalence by Province, Small Area Composite Method

**Province**	**Total**		**Male**		**Female**	
	**Proportion (95% CI)**	**MSE**	**Proportion (95% CI)**	**MSE**	**Proportion (95% CI)**	**MSE**
A-gharbi	0.244 (0.209-0.279)	0.00031	0.196 (0.167-0.226)	0.00022	0.293 (0.172-0.413)	0.00376
A-sharghi	0.243 (0.211-0.275)	0.00027	0.216 (0.16-0.272)	0.00081	0.271 (0.214-0.328)	0.00083
Alborz	0.259 (0.214-0.303)	0.00052	0.251 (0.08-0.422)	0.00761	0.266 (0.214-0.317)	0.00069
Ardebil	0.214 (0.155-0.272)	0.00089	0.193 (0.149-0.238)	0.00052	0.235 (0.151-0.318)	0.00183
Booshehr	0.227 (0.147-0.307)	0.00167	0.22 (0.129-0.311)	0.00215	0.233 (0.147-0.318)	0.00190
Chaharmahal- va-bakhtiari	0.196 (0.081-0.312)	0.00346	0.167 (0.06-0.274)	0.00297	0.225 (0.126-0.325)	0.00258
Fars	0.265 (0.19-0.34)	0.00147	0.235 (0.135-0.334)	0.00258	0.295 (0.17-0.421)	0.00411
Ghazvin	0.274 (0.174-0.373)	0.00258	0.244 (0.102-0.387)	0.00528	0.305 (0.125-0.484)	0.00843
Ghom	0.245 (0.173-0.316)	0.00133	0.228 (0.146-0.311)	0.00177	0.262 (0.202-0.321)	0.00092
Gilan	0.241 (0.202-0.279)	0.00038	0.192 (0.156-0.228)	0.00033	0.288 (0.184-0.391)	0.00279
Golestan	0.248 (0.21-0.285)	0.00036	0.229 (0.111-0.347)	0.00362	0.266 (0.219-0.313)	0.00057
Hamedan	0.227 (0.179-0.274)	0.00059	0.213 (0.148-0.278)	0.00111	0.241 (0.185-0.297)	0.00081
Hormozgan	0.267 (0.164-0.37)	0.00276	0.235 (0.099-0.371)	0.00481	0.301 (0.147-0.454)	0.00613
Ilam	0.171 (0.087-0.256)	0.00184	0.152 (-0.043-0.347)	0.00987	0.192 (-0.079-0.463)	0.01911
Isfahan	0.259 (0.207-0.312)	0.00072	0.236 (0.14-0.333)	0.00242	0.283 (0.19-0.376)	0.00225
Kerman	0.223 (0.185-0.261)	0.00038	0.203 (0.17-0.236)	0.00028	0.243 (0.191-0.295)	0.00070
Kermanshah	0.244 (0.196-0.291)	0.00058	0.223 (0.15-0.296)	0.00138	0.264 (0.215-0.313)	0.00063
Khorasan jonoobi	0.238 (0.152-0.324)	0.00191	0.196 (0.147-0.244)	0.00061	0.278 (0.187-0.368)	0.00212
Khorasan razavi	0.208 (0.125-0.292)	0.00180	0.182 (0.124-0.241)	0.00089	0.234 (0.164-0.304)	0.00126
Khorasan shomali	0.223 (0.155-0.292)	0.00122	0.218 (0.116-0.321)	0.00271	0.228 (0.124-0.332)	0.00282
Khozestan	0.224 (0.191-0.258)	0.00029	0.2 (0.177-0.222)	0.00013	0.25 (0.221-0.278)	0.00020
Kohgiloyeh-va- boyer-ahmad	0.2 (0.084-0.315)	0.00348	0.181 (0.106-0.255)	0.00143	0.22 (0.065-0.375)	0.00629
Kurdistan	0.215 (0.154-0.276)	0.00098	0.188 (0.144-0.232)	0.00051	0.242 (0.187-0.297)	0.00079
Lorestan	0.183 (0.026-0.339)	0.00636	0.148 (0.002-0.295)	0.00561	0.217 (0.099-0.335)	0.00362
Markazi	0.236 (0.183-0.29)	0.00074	0.211 (0.16-0.262)	0.00067	0.262 (0.213-0.311)	0.00062
Mazandaran	0.219 (0.178-0.26)	0.00044	0.192 (0.163-0.222)	0.00023	0.245 (0.211-0.279)	0.00030
Semnan	0.364 (-0.144-0.871)	0.06707	0.314 (-0.139-0.767)	0.05339	0.415 (-0.245-1.075)	0.11352
Sistan-va- balouchestan	0.266 (0.176-0.356)	0.00212	0.201 (0.164-0.238)	0.00036	0.329 (0.115-0.544)	0.01196
Tehran	0.231 (0.201-0.262)	0.00025	0.203 (0.178-0.228)	0.00016	0.26 (0.227-0.293)	0.00028
Yazd	0.234 (0.161-0.308)	0.00141	0.185 (0.121-0.248)	0.00105	0.288 (0.169-0.407)	0.00370
Zanjan	0.319 (0.043-0.596)	0.01995	0.279 (0.004-0.554)	0.01963	0.36 (-0.027-0.748)	0.03917

**Table 8:** Psychiatric Disorder Prevalence by Province, Small Area Hierarchical Bayes Method

**Province**	**Total**		**Male**		**Female**	
	**Proportion (CI)**	**MSE**	**Proportion (CI)**	**MSE**	**Proportion (CI)**	**MSE**
A-gharbi	0.245 (0.2-0.291)	0.00052	0.184 (0.135-0.239)	0.00071	0.314 (0.249-0.383)	0.00116
A-sharghi	0.224 (0.184-0.264)	0.00043	0.201 (0.154-0.253)	0.00065	0.255 (0.201-0.314)	0.00083
Alborz	0.277 (0.217-0.337)	0.00052	0.287 (0.204-0.384)	0.00213	0.269 (0.2-0.347)	0.00140
Ardebil	0.178 (0.12-0.236)	0.00081	0.177 (0.112-0.254)	0.00132	0.215 (0.141-0.3)	0.00163
Booshehr	0.234 (0.166-0.303)	0.00128	0.203 (0.129-0.29)	0.00168	0.273 (0.19-0.369)	0.00207
Chaharmahal- va-bakhtiari	0.166 (0.107-0.225)	0.00096	0.158 (0.09-0.237)	0.00143	0.215 (0.142-0.298)	0.00160
Fars	0.281 (0.241-0.321)	0.00038	0.25 (0.2-0.304)	0.00070	0.318 (0.263-0.377)	0.00084
Ghazvin	0.252 (0.186-0.318)	0.00118	0.233 (0.162-0.316)	0.00156	0.266 (0.184-0.359)	0.00198
Ghom	0.262 (0.193-0.331)	0.00127	0.227 (0.152-0.316)	0.00174	0.293 (0.211-0.386)	0.00202
Gilan	0.23 (0.182-0.279)	0.00060	0.175 (0.12-0.237)	0.00091	0.294 (0.229-0.363)	0.00117
Golestan	0.253 (0.194-0.312)	0.00092	0.237 (0.164-0.325)	0.00169	0.273 (0.203-0.351)	0.00142
Hamedan	0.201 (0.147-0.254)	0.00079	0.201 (0.134-0.28)	0.00138	0.202 (0.138-0.276)	0.00125
Hormozgan	0.258 (0.197-0.318)	0.00097	0.213 (0.146-0.292)	0.00139	0.299 (0.224-0.382)	0.00162
Ilam	0.175 (0.109-0.241)	0.00013	0.152 (0.076-0.241)	0.00179	0.187 (0.092-0.27)	0.00021
Isfahan	0.284 (0.245-0.323)	0.00037	0.27 (0.22-0.324)	0.00072	0.303 (0.252-0.358)	0.00074
Kerman	0.211 (0.169-0.253)	0.00037	0.21 (0.152-0.275)	0.00098	0.212 (0.16-0.27)	0.00079
Kermanshah	0.252 (0.197-0.306)	0.00058	0.246 (0.179-0.323)	0.00135	0.268 (0.199-0.344)	0.00137
Khorasan jonoobi	0.26 (0.179-0.342)	0.00182	0.203 (0.124-0.301)	0.00201	0.304 (0.209-0.415)	0.00278
Khorasan razavi	0.159 (0.13-0.188)	0.00022	0.139 (0.101-0.182)	0.00044	0.196 (0.157-0.237)	0.00042
Khorasan shomali	0.205 (0.14-0.27)	0.00117	0.206 (0.128-0.304)	0.00199	0.192 (0.117-0.278)	0.00168
Khozestan	0.195 (0.159-0.231)	0.00023	0.191 (0.146-0.24)	0.00058	0.218 (0.169-0.272)	0.00070
Kohgiloyeh-va- boyer-ahmad	0.184 (0.119-0.249)	0.00120	0.161 (0.092-0.242)	0.00149	0.202 (0.119-0.298)	0.00208
Kurdistan	0.188 (0.134-0.243)	0.00071	0.179 (0.119-0.246)	0.00104	0.228 (0.154-0.311)	0.00160
Lorestan	0.126 (0.085-0.166)	0.00047	0.119 (0.068-0.177)	0.00080	0.185 (0.124-0.252)	0.00108
Markazi	0.205 (0.148-0.261)	0.00072	0.188 (0.121-0.267)	0.00141	0.242 (0.172-0.318)	0.00142
Mazandaran	0.201 (0.162-0.24)	0.00039	0.183 (0.127-0.247)	0.00093	0.205 (0.157-0.259)	0.00069
Semnan	0.365 (0.254-0.476)	0.00321	0.345 (0.223-0.496)	0.00499	0.384 (0.265-0.524)	0.00443
Sistan-va- balouchestan	0.3 (0.249-0.351)	0.00068	0.197 (0.142-0.258)	0.00088	0.378 (0.308-0.451)	0.00134
Tehran	0.194 (0.166-0.222)	0.00018	0.165 (0.131-0.203)	0.00034	0.238 (0.198-0.28)	0.00044
Yazd	0.248 (0.178-0.319)	0.00135	0.211 (0.133-0.307)	0.00196	0.289 (0.207-0.383)	0.00202
Zanjan	0.361 (0.28-0.442)	0.00170	0.274 (0.187-0.383)	0.00252	0.388 (0.29-0.495)	0.00276

**Appendix 3**
